# Effect of Plastic Deformation on the Structure and Mechanical Properties of the Zn-4Ag-1Cu Zinc Alloy

**DOI:** 10.3390/ma16134646

**Published:** 2023-06-27

**Authors:** Elvira Khafizova, Elvira Fakhretdinova, Rinat Islamgaliev, Milena Polenok, Vil Sitdikov, Hakan Yilmazer

**Affiliations:** 1 Institute of Physics of Advanced Materials, Ufa University of Science and Technology, 450076 Ufa, Russia; 2 Institute of Molecules and Crystal Physics, Ufa Federal Research Center of the Russian Academy of Sciences, 450075 Ufa, Russia; 3 LLC “RN-BashNIPIneft”, Lenina Street 86/1, 450076 Ufa, Russia; 4 Department of Metallurgical and Materials Engineering, Yildiz Technical University, 34220 Esenler, Istanbul, Turkey; 5 Health Biotechnology Joint Research and Application Center of Excellence, 34220 Esenler, Istanbul, Turkey

**Keywords:** biodegradable metals, Zn-based alloys, mechanical properties, microstructures, equal-channel angular pressing, computer simulation

## Abstract

It is known that zinc biodegradable alloys are a promising material for producing biomedical implants for orthopedics and vascular stents. Among them, the Zn-Ag-Cu zinc alloy is of special interest due to the antibacterial and antimicrobial properties of Ag and Cu. To improve the mechanical properties of the Zn-4Ag-1Cu zinc alloy, the effect of equal-channel angular pressing (ECAP) on the microstructure and strength has been investigated. The ECAP conditions for the Zn-4Ag-1Cu alloy were chosen by modeling in the Deform 3 D program (temperature and strain rate). The microstructure was analyzed using transmission electron microscopy, scanning electron microscopy and X-ray diffraction analysis. The study of strength was carried out by measuring the microhardness and tensile tests of small samples with a gauge dimension of 0.8 × 1 × 4 mm^3^. The microstructure after ECAP was characterized by equiaxed grains ranging in a size from 1.5 µm to 4 µm with particles in a size from 200 nm to 1 µm uniformly distributed along the boundaries. The ECAP samples showed a high strength of 348 MPa and good ductility of up to 30%, demonstrating their great potential as promising materials for producing medical stents.

## 1. Introduction

Currently, much attention in medicine is paid to the problem of traumatic injuries of bones and soft tissues, which increases the need for a biocompatible material that is non-toxic during prolonged exposure to the human body. The current direction of research is the development of new biodegradable materials that have the ability to completely dissolve in the body over a certain period of time, which eliminates the need for repeated operations [1,2].

Among them, biodegradable metals based on Mg, Fe and Zn are of great interest. In particular, zinc-based alloys are new and very promising biodegradable materials for producing medical implants [3,4]. Zn is known to have the ideal corrosion rate among biodegradable alloys [5,6]. In addition, Zn promotes cell reproduction by dividing and is indispensable in the immune and nervous systems [7]. Although Zn has a lower toxicity limit (100–150 mg/day) than Mg (375–500 mg/day), this does not restrict the use of Zn in biodegradable implants. The main condition is that during corrosion, metal ions should not exceed the toxicity limit per day. All this information indicates that Zn is a promising material for producing biodegradable implants. However, despite its advantages, the main disadvantage of zinc is its low strength. It is known that the ultimate tensile strength (UTS) of cast Zn is less than 50 MPa [1,5,8]. Therefore, improving the strength of pure Zn is an important task.

Among the methods to improve the mechanical properties of Zn is its alloying [9]. However, the number of alloying elements that can be added to pure Zn without reducing corrosion resistance and biocompatibility is limited [1,10]. Zn alloyed with Ag and Cu has antibacterial and antimicrobial properties, which is ideal for urinary stents. Many studies based on the antibacterial properties of Ag confirm that Ag ions kill bacteria adhering to the implant surface or prevent them from adhering to the implant surface [1]. Cu is also an essential microelement required for the development of connective tissues and nerve sheaths, as well as for bone growth [11]. In addition, the Cu ion is well known to have antimicrobial properties [12].

Another approach to improving mechanical properties is grain refinement by hot extrusion, rolling or severe plastic deformation (SPD) [13] based on the use of large plastic deformations at elevated pressures and relatively low temperatures [14,15].

For example, it is known that the use of hot extrusion increases the UTS of pure Zn up to 110 MPa with an increase in ductility up to 14% [16]. Another study [17] showed that hot rolling increases the strength and ductility of pure Zn to 118 MPa and 26.8%, respectively. However, this level of strength is still insufficient for medical applications starting from 300 MPa [7]. Application of high-pressure torsion has shown that the grain refinement of pure Zn to the nanoscale increases the UTS of the material up to 260 MPa and ductility up to 40% [18,19]. It has been shown that high-pressure torsion also significantly strengthens Zn-0.5%Cu [20] and Zn-0.8%Ag [21] alloys. At the same time, the increase in mechanical properties is not accompanied by a decrease in the corrosion resistance of pure Zn [19].

It has been shown that application of one of the efficient SPD methods, namely the equal-channel angular pressing (ECAP), to the Zn-0.1% Mg alloy leads to an increase in UTS up to 383 MPa with an elongation of 45.6% [22]. For example, it was shown that after 2 ECAP passes at 200 °C, the grain size decreased from 48 mm in the as-cast alloy to 1.8 μm after ECAP, and there was a large increase in YS, UTS and elongation from 65 MPa, 84 MPa and 1.3% (in the as-cast alloy) to 205 MPa, 220 MPa and 6.3%, respectively [23].

Investigations of three hot extruded Zn-Ag alloys with an Ag content from 2.5 to 7.0 wt.% showed that an increase in the Ag content increases UTS, but does not significantly affect the ductility [24]. The Zn–7.0%Ag alloy exhibited enhanced YS and UTS (236 and 287 MPa, respectively), which is associated with grain refinement and a large volume fraction of the fine AgZn_3_ particles precipitating along the grain boundaries during extrusion.

However, there are studies where grain refinement by ECAP does not confirm the Hall–Petch relation that can be associated with activation of the creep deformation mechanisms, namely grain boundary sliding in low-alloyed Zn-based materials [25].

Although the Zn-based alloys were researched in the literature, there are no systematic studies directly on ultrafine-grained biodegradable Zn-Ag-Cu alloys. Thus, a new Zn-4Ag-1Cu alloy processed by ECAP to produce ultarfine-grained structure has been investigated. To obtain defect-free ultrafine-grained samples, the temperature and deformation modes of ECAP processing were elaborated using computer simulation. Phase transformations after ECAP processing were studied using SEM, TEM and X-ray diffraction. The microstructural features leading to enhanced strength of the ECAP samples were established.

## 2. Experimental Section

### 2.1. Processing

The Zn-Ag-Cu alloy was smelted in a chamber furnace. The temperature in the furnace was 580 °C. After complete dissolution and prior to casting, the metal was mixed. The metal was cast into a detachable metal mold 20 mm in diameter, heated to 150 °C. The chemical composition was determined using the Q4 TASMAN (Bruker, Germany) optical emission spectrometer (Table 1).

Homogenization with subsequent water quenching was carried out at 400 °C for 24 h. The heat treatment of the samples was conducted in a Nabertherm muffle furnace (Germany).

**Figure 1:**
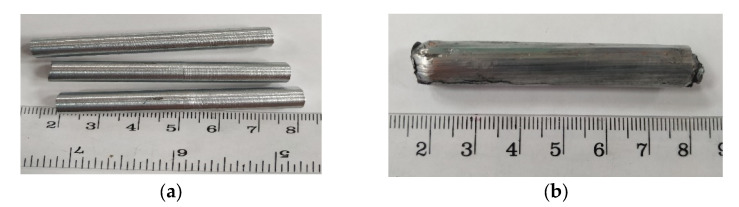
Appearance of samples: (**a**) after rolling and machining up to Ø10 mm, (**b**) Ø10 mm after ECAP.

Initial samples of Ø20 mm were rolled at a temperature of 200 °C to Ø14 mm on a Hankook M-tech six-high rolling mill (Korea) and then machined to the required diameter 10 mm (Figure 1).

The ECAP billet was repeatedly pressed in a special die set through two channels with identical cross sections intersecting at an angle of 120°. To perform ECAP processing, a press with a force of 160 ton and the universal die were used. The die was equipped with a furnace with an accuracy of ±5 °C. Prior to each pass, the billets were lubricated with a graphite-containing lubricant. The billets were subjected to deformation at a temperature and deformation speed selected using the results of computer simulation. Root Bc (after each pass the workpiece was rotated around its longitudinal axis by 90° clockwise) and 4 cycles at an 120° intersecting angle of channels were used.

To preheat the workpieces, a SNOL furnace was used, in which the samples were heated for 20 min at a temperature equal to the deformation temperature.

Samples for study after ECAP processing were cut parallel to the transverse and longitudinal sections from the central part of the workpieces.

### 2.2. Simulation

To simulate the processing modes of ECAP, the software package Deform-3D v.11 was chosen, designed to analyze the three-dimensional flow of metal by the finite element method, which is often used in the analysis of metal forming processes. This program allows to analyze the behavior of the metal, the temperature field, the load on the tool in a 3D format during various metal forming operations. The workpiece and tool models were created using the CAD system—KOMPAS-3D. The workpiece is a plastic body, the die tool is an absolutely rigid body. The impenetrability condition was set on the contact surfaces of the tooling. For the case of modeling a three-dimensional deformation scheme with high contact stresses, the Siebel friction factor was used.

**Figure 2:**
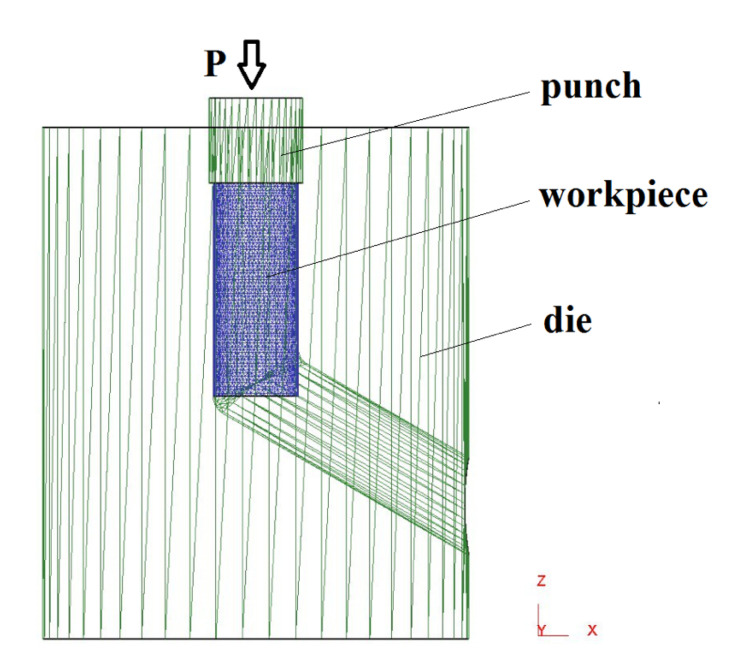
Scheme of the ECAP process.

When modeling in the Deform-3D, the workpiece was divided into tetrahedra, the number of which was 64,000–78,500, and the size of one element was 0.54 mm. The scheme of the ECAP process adopted in the simulation is shown in Figure 2.

In the course of the research, the following variants of deformation modes were analyzed: (a) processing temperature T = 150 °C, deformation speed v = 0.4 mm/s; (b) T = 200 °C, v = 0.4 mm/s; (c) T = 200 °C, v = 7.8 mm/s; (d) T = 150 °C, v = 7.8 mm/s. The deformation speeds were chosen as minimum (v = 0.4 mm/s) and maximum (v = 7.8 mm/s) for the available equipment—a press with a force of 160 ton. The processing temperatures (20, 150, 200, 250 °C) were chosen based on the mechanical properties of the zinc alloy at various temperatures and strain rates (Table 2).

To analyze the homogeneity of the stress fields, the values of the accumulated strain in the cross-section of the workpiece were calculated. To evaluate the implemented principle (compression–tension), the values of average stresses were analyzed. To analyze the homogeneity of the structure and mechanical properties, the values of strain rates at shear sites were studied. In addition, the maximum values of temperatures and force parameters were estimated. The analysis criteria took into account the following circumstances:

- Increased values of tensile stresses in the ECAP-treated specimens lead to the initiation of cracks;
- Due to internal friction, deformation heating increases, and with an increase in speed, the process of grain refinement is intensified due to an increase in the density of dislocations and formation of low- and high-angle grain boundaries. Accordingly, the increased non-uniformity of the strain rate distribution affects the uniformity of the structure and mechanical properties of the workpieces;
- The maximum values of force parameters are the main condition for choosing tooling and tool material.

The uniformity of deformations was studied by evaluating the revealed difference in the accumulated strains in the cross section of the sample.

The computer simulation assumptions were as follows:

- Material of the initial billet—zinc alloy Zn-Ag-Cu;
- Dimensions of the original workpiece d = 10 mm, length 60 mm;
- Number of finite elements 64,000–78,500, element size 0.54 mm;
- Conditions for compensating the volume of the workpiece model are included;
- Deformation temperature 150, 200 °C;
- Friction coefficient is equal to µ = 0.3;
- Number of simulation steps 100–250 with a step of 0.5 s;
- Deformation speed v = 0.4, v = 7.8 mm/s.

### 2.3. Microstructural Characterization

The microstructure of the alloy was studied using an FEI Thermo Scientific Q250 scanning electron microscope (SEM) at an accelerating voltage of 25 kV and a JEM-2100 transmission electron microscope (TEM) operating at an accelerating voltage 200 kV. Investigations into microstructure in SEM were carried out using an Everhart–Thornley detector with secondary electrons. To study the microstructure of the ECAP samples on SEM and TEM, thin foils were obtained on a Tenupol-5 device using double jet electropolishing at a temperature of −35 °C and a voltage of 20 V in an electrolyte consisting of 95% ethanol and 5% perchloric acid.

X-ray diffraction analysis was performed on a Rigaku Ultima IV diffractometer. Diffraction patterns were recorded in the continuous scanning mode at a speed of 1°/min within the scattering angle of 2θ from 10° to 150° on Cu radiation generated at a voltage of 40 kV and a current of 40 mA. Qualitative X-ray phase analysis (XRF) was performed via the EVAplus program using the PDF-2 X-ray database. Quantitative phase analysis was performed by the Rietfeld method in the TOPAS v. 4.2 (www.bruker.com, accessed on 22 June 2023).

### 2.4. Mechanical Performance

**Figure 3:**
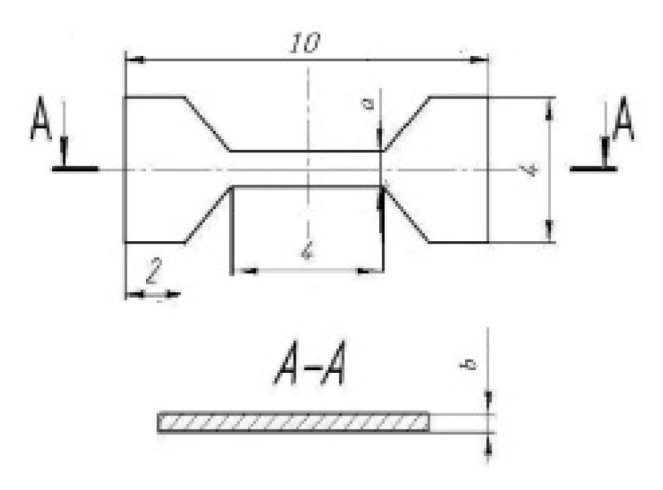
Scheme of specimens for tensile tests.

Tensile tests were conducted on an Instron 5982 testing machine at various strain rates (0.1, 0.5 and 1 s^−1^) and temperatures (20, 150, 200, 250 °C) using small samples with a gauge dimension of 0.8 × 1 × 4 mm (Figure 3). Tensile properties such as YS (σ_02_), UTS (σ_UTS_) and elongation to failure (δ) were evaluated by testing 3 samples in each condition to ensure reproducible results.

Hv microhardness was measured by the Vickers method using a Buehler Micromet 5101 facility with a pyramid-shaped diamond indenter under a load of 0.1 kg for 10 s according GOST 9450-60.

## 3. Results

### 3.1. Simulation

The hardening curves of the initial Zn alloy samples were entered into the Deform-3D library based on the results of tensile tests at various strain rates (0.1, 0.5 and 1 s^−1^) and temperatures (20, 150, 200, 250 °C) (Table 2).

**Figure 4:**
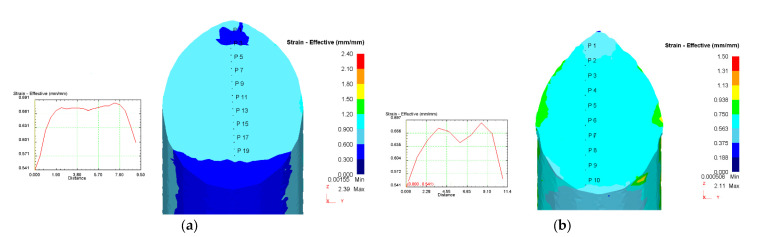
Strain distribution in the cross section of the workpiece: (**a**) T = 150 °C, v = 0.4 mm/s; (**b**) T = 200 °C, v = 7.8 mm/s.

The distribution of the accumulated strain during processing at various temperatures and deformation speeds is shown in Figure 4. Strain distribution over the section under various processing conditions is generally uniform; the difference between the minimum and maximum values is about 0.15–0.2. The curve of change in the accumulated strain has a parabolic character for two options: T = 150 °C, v = 0.4 mm/s; and T = 200 °C, v = 7.8 mm/s (Figure 4). In the central part of the workpiece, strains are more uniform and reach a maximum value of 0.7, which is typical for ECAP processing with a channel intersecting angle of 120° [7]. Consequently, the temperature and deformation speed in the studied range of specified parameters do not affect the strain distribution during ECAP of zinc alloy.

**Figure 5:**
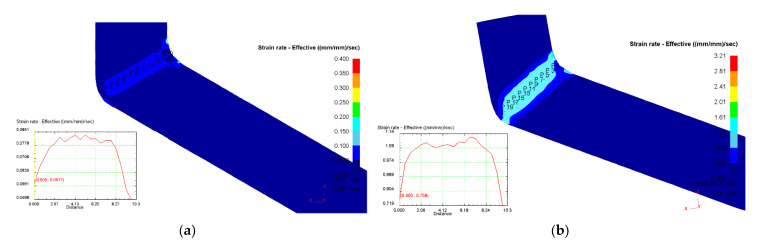
Strain rate distribution in the longitudinal section of the workpiece: (**a**) T = 200 °C, v = 0.4 mm/s; (**b**) T = 150 °C, v = 7.8 mm/s.

From the analysis of the strain rate fields in the deformation zones for the given pressing conditions, it can be seen that the strain rate values vary in the range of 0.05–0.08 at the deformation speed v = 0.4 mm/s (Figure 5a) and in the range of 0.8–1.06 at the deformation speed v = 7.8 mm/s (Figure 5b). This confirms that a decrease in the deformation speed leads to more uniform strain deformation in the deformation zones of the metal during ECAP processing.

**Figure 6:**
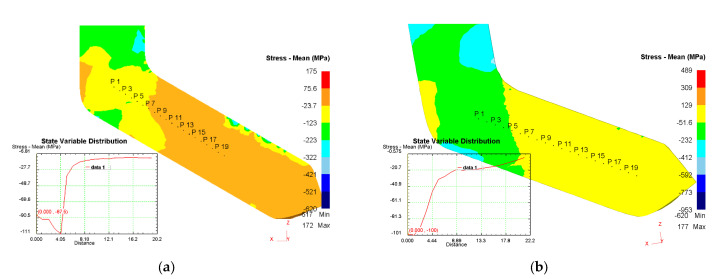
Average stress distribution in the longitudinal section of the workpiece: (**a**) T = 200 °C, v = 0.4 mm/s; (**b**) T = 150 °C, v = 7.8 mm/s.

Figure 6 shows the stress distribution patterns which can be used to determine the values of compressive and tensile stresses forming on the workpiece surface in one cycle of ECAP processing. As can be seen from Figure 6, compressive stresses mainly act on the surface of the samples while there are practically no tensile stresses in the deformation zone, so it is possible to predict the production of defect-free workpieces under all of studied temperature and deformation speed conditions.

The maximum values of the billet heating temperature after one pass of ECAP and deformation forces were also estimated. The results of the research are presented in Table 3.

From the analysis of the results obtained, it was found that an increase in the deformation speed at the same processing temperatures leads to an increase in the load by about 5%. An increase in temperature from 150 to 200 °C at the same deformation speeds causes insignificant changes.

The maximum heating of the workpiece (the difference between the initial and maximum temperatures) is observed with an increase in the deformation speed; therefore, an increase in the deformation speed entails a greater heating of the workpiece.

### 3.2. Microstructure

**Figure 7:**
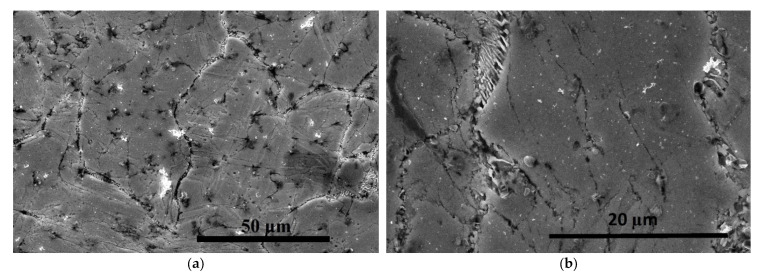
SEM-SEI microstructure after rolling: (**a**) along the rolling direction, (**b**) across the rolling direction.

The cast structure of the Zn alloy consists of primary crystals and eutectics, in which an increased concentration of Ag is observed. The microstructure after rolling is shown in Figure 7, where the eutectic plates are visible along the grain boundaries. In the longitudinal section, the orientation of the structural elements along the rolling direction is observed. Inside the eutectics, a mix of phases is observed in the form of plates with sizes reaching 700 nm in width.

**Figure 8:**
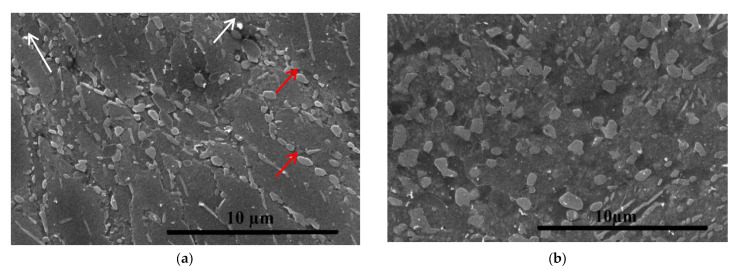
SEM-SEI microstructure after ECAP: (**a**) at 150 °C; (**b**) at 200 °C; arrows show particles.

In the samples subjected to ECAP at 150 °C for four passes, a banded structure with an average width of 1.3 µm (Figure 8a) containing two different types of precipitates ranging in size from 90 to 690 nm is observed.

**Figure 9:**
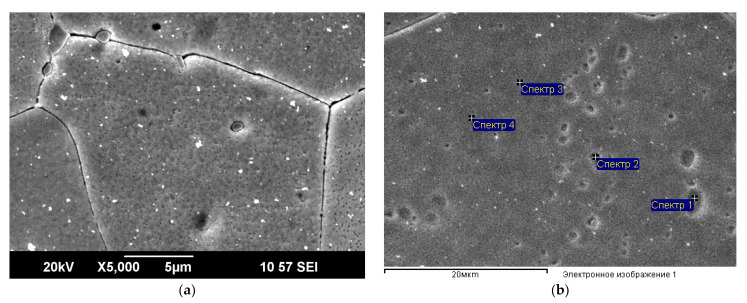
(**a**) SEM-SEI microstructure after ECAP at 150 °C and annealing at 350 °C: (**b**) points selected for EDS analysis in Table 4.

After ECAP at 200 °C, there is some coarsening of precipitates and grain growth up to 2.4 µm (Figure 8b). According EDS analysis (Table 4, Figure 9b), globular grey particles of 690 mm in diameter contain Ag and Cu over the content in the chemical composition of the material (Table 1). The light Ag-rich particles of 90 nm in a size do not contain Cu higher than in the chemical composition of the material (Table 1 and Table 4).

Grain growth up to 30 µm and two different types of precipitates after additional annealing at a temperature of 350 °C were observed (Figure 9). From EDS analysis (Table 4), one can assume that large grey particles contain Zn, Cu and Ag, whereas small light particles are composed of Zn and Ag.

**Figure 10:**
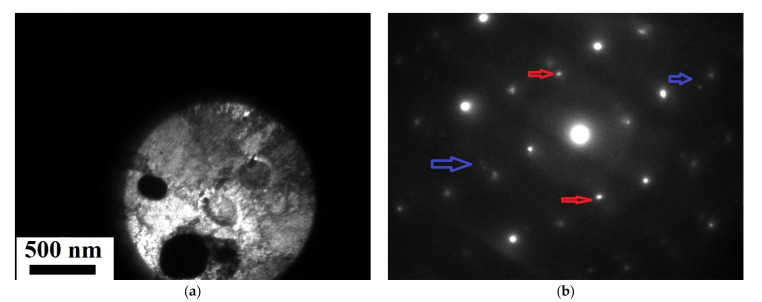
TEM microstructure after ECAP: (**a**) bright-field image of particles, (**b**) electron diffraction pattern from this particle.

TEM studies do not provide information on grain sizes, but we can observe particles the spots from which on the electron diffraction pattern belong to AgZn3 particles (Figure 10).

**Figure 11:**
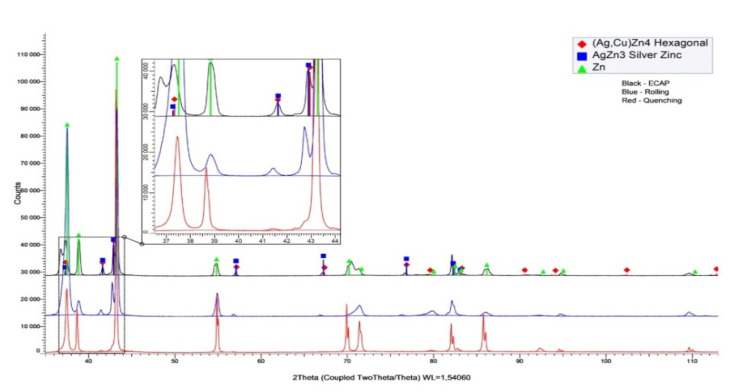
View of the X-ray diffraction patterns of the Zn alloy.

To study phase transformations, an X-ray diffraction analysis of the alloy was carried out after various processing modes. Figure 11 shows a general view of the X-ray diffraction patterns of the Zn alloy subjected to quenching, rolling and ECAP at 150 °C.

Qualitative XRD showed that only (AgCu)Zn4 [26] particles were observed after rolling (Figure 11). At the same time, ECAP treatment at a temperature of 150 °C leads to an increase in the fraction of (AgCu)Zn4 precipitates, additional precipitation of AgZn3 particles and increase in dislocation density (Table 5). One can also see the presence of two different precipitates after ECAT at 150 °C in Figue 8a. One can note that crystal lattices of all three phases Zn, (AgCu)Zn_4_ and AgZn_3_ are distorted in comparison with tabular data. For example, we observed in the quenched samples of Zn the experimental parameters of crystal lattice a = 2.6844 and c = 4.7983 in comparison with tabular data a = 2.6636 and c = 4.9457 from PDF files.

### 3.3. Mechanical Properties

**Figure 12:**
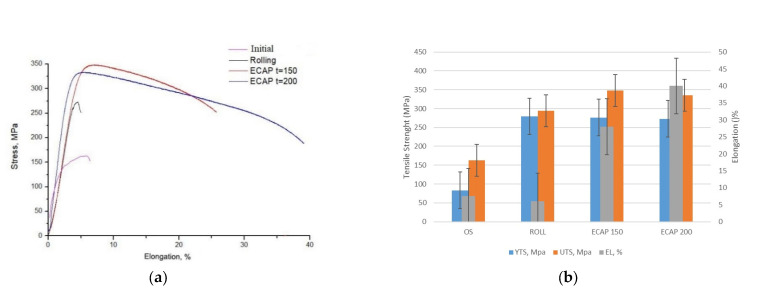
Tensile properties after various treatments: (**a**) typical engineering stress–engineering strain curves; (**b**) summary of YS, UTS and EL.

**Figure 13:**
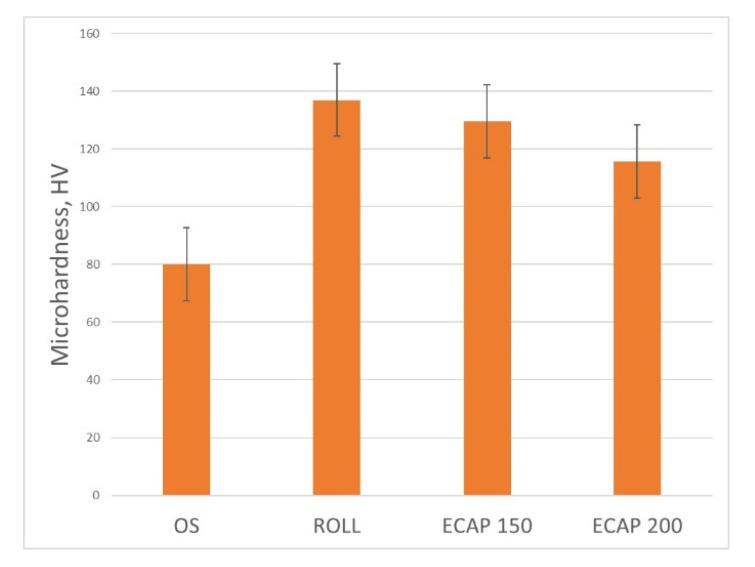
Microhardness after various treatments.

The ultimate tensile strength (UTS) of samples processed by ECAP at 150 °C has a maximum of 348 MPa at a ductility of 27% (Figure 12). After ECAP at 200 °C, the UTS slightly decreases to 331 MPa with increasing ductility up to 38%. Microhardness of the rolled samples has the highest value in the cross-section equal to 139 HV (Figure 13).

To study the thermal stability of the ECAP samples, annealing was performed for one hour at various temperatures (Table 6).

In the course of studies of thermal stability up to a temperature of 350 °C, the microhardness of the ECAP samples practically did not change, which can indicate an effectiveness of dispersion hardening during heating of the material.

## 4. Discussion

According to the results of computer simulation, it was found that the temperature and strain rate in the studied range of parameters do not have a noticeable effect on strain distribution during the ECAP of zinc alloy (Figure 4). This corresponds to the opinion that strain distribution during ECAP is determined mainly by the geometry of the channels, the angle of their intersection, tribological conditions and the strain capacity [14,27,28].

It was also found that a decrease in the deformation speed leads to more uniform strain distribution in the metal deformation zone during ECAP (Figure 5). A relatively low deformation speed is important for the occurrence of relaxation processes and improving plasticity of metals during SPD.

On the surface of the samples, compressive stresses mainly act (Figure 6), and tensile stresses in the deformation zone are practically absent. Therefore, it is possible to predict the production of defect-free workpieces under various temperature and deformation speeds. In this case, special attention should be paid to the near-contact layer in the outer corner of the intersection of the channels, where the occurrence of tensile stresses is more likely. In the work conducted, an increase in the deformation speed led to enhanced compressive stresses (Figure 6), so low deformation speed was selected for ECAP processing.

Thus, according to results of computer simulation, to obtain defect-free samples from the Zn-4Ag-Cu alloy, ECAP processing at low deformation speed v = 0.4 mm/s and temperatures of 150 and 200 °C was chosen.

It is known that the addition of Ag and Cu to zinc simultaneously increases the uniform elongation during tensile tests [29]. Additionally, ductility of the Zn-Cu alloy can increase after the grain size decreasing below 1 µm [29]. In this case, due to low melting temperature, the interfacial slip can be activated, as well as the grain boundary sliding, leading to enhanced plasticity.

The increase in strength of the ECAP samples can be attributed to a combination of several strengthening mechanisms including grain refinement, dispersion hardening, dislocation hardening and solid solution strengthening.

Generally, contribution of grain boundary strengthening σ_gb_ is expressed by the conventional Hall–Petch relationship [30],

σ_gb_ = σ_o_ + kd^−1/2^, (1)

where *σ_o_* represents the friction stress for pure Zn equal to 11 MPa [31], *d* is the mean grain size, *k* is the coefficient for pure Zn taken as 0.22 MPa × m^1/2^ [30]. The grain boundary strengthening *kd^−^*^1/2^ for each alloy is calculated as 193 MPa for ECAP 150 and 142 MPa for ECAP 200.

The dislocation hardening is calculated using the Bailey–Hirsch relation [32],

(2) $$\sigma_{d}=\alpha MbG\rho^{1/2},$$

where *α* = 0.2 is a constant, *M =* 3.06 is the Taylor factor, *b* = 0.26649 nm is the Burgers vector, G = 37.7 GPa is the shear modulus, *ρ* is the dislocation density (10^14^ m^−2^ for ECAP 150 and 10^13^ m^−2^ for ECAP 200). The dislocation strengthening is 66 MPa for ECAP 150 and 39 MPa for ECAP 200.

Solid solution strengthening *σ_ss_* can determined by a concentration of solutes [33]:

*σ_ss_ = k_i_ c^n^_i,_* (3)

where *k_i_* is the strengthening coefficient; *c_i_* is the concentration of solute *i*, at. pct.

Taking into account the averaged coefficient *k_i_* = 111.8 and *n* = 0.52 for Zn alloys [31] and the chemical composition of the alloy (Table 1), in the first approximation, the solid solution strengthening is calculated to be equal 16 MPa for both samples ECAP 150 and ECAP 200.

For calculation of precipitation strengthening *σ_pp_*, we used the Ashby–Orowan relationship [34]

(4) σpp=0.538Gbf12d−1lnd2b,

where *G* = 37.7 GPa is the shear modulus, *b* = 0.26649 nm is the Burgers vector, *f*, *d* is the volume fraction and size of precipitates (Table 5).

The calculated contributions from strengthening mechanisms to strength are presented in Table 7. Analysis of strengthening mechanisms confirms that yield stress of both ECAP samples is ensured mainly by grain boundaries and additionally by precipitates and dislocations.

Thus, it has been shown that the use of ECAP at temperatures of 150 °C and 200 °C leads to grain refinement and an increase in the fraction of the (ACu)gZn_4_ and AgZn_3_ precipitates. The selected processing conditions made it possible to achieve enhanced strength and ductility, which are of interest for the use of this alloy as a material for producing biodegradable medical implants.

## 5. Conclusions

To optimize the ECAP parameters of the new Zn-4Ag-1Cu alloy, computer simulation in the Deform 3D program was used. The effect of various ECAP parameters on microstructure, tensile properties and Vicker’s microhardness has been thoroughly examined; the results are summarized below.

1. According to results of computer simulation, it is recommended to carry out ECAP of the Zn-4Ag-1Cu alloy with a low deformation speed of 0.4 mm/s and temperatures of 150 °C and 200 °C.
2. The grain refinement and additional precipitation of (AgCu)Zn_4_ and AgZn_3_ in the ECAP samples leads to enhanced ultimate tensile strength of 348 MPa at a ductility of 27%. For comparison, 195 MPa and 13%, respectively, were observed in the initial coarse-grained samples. Yield stress of both ECAP samples is ensured by grain boundaries and additionally by precipitates and dislocations.
3. The microhardness of the ECAP samples remains stable after heating to temperature of 350 °C, although the grain size increases to 30 μm. This may indicate a violation of the correlation between microhardness and a grain size during heating of ECAP samples as a result of increasing fraction of precipitates.

## Figures and Tables

**Table 1:** Chemical composition, wt.%.

Zn	Ag	Cu	Mg
93.86 ± 0.1	3.89 ± 0.02	1.02 ± 0.08	0.44 ± 0.02

**Table 2:** Results of tensile tests at various strain rates and temperatures in Zn-4Ag-Cu alloy.

Temperature, °C	Strain Rate, s^−1^	σ_0_._2_, MPa	σ_UTS_, MPa	True Strain
20	0.1	83.16	163.06	0.05
	0.5	140.67	198.24	0.12
	1.0	105.16	138.50	0.05
150	0.1	126.51	186.03	0.14
	0.5	81.20	181.05	0.10
	1.0	182.63	126.33	0.16
200	0.1	115.43	161.27	0.15
	0.5	88.79	164.79	0.12
	1.0	99.12	173.17	0.16
250	0.1	102.34	129.03	0.12
	0.5	118.85	148.61	0.16
	1.0	48.48	138.89	0.12

**Table 3:** Results of the study of the force parameters and temperature.

Parameters	T = 150 °C, v = 0.4 mm/s	T = 150 °C, v = 7.8 mm/s	T = 200 °C v = 0.4 mm/s	T = 200 °C, v = 7.8 mm/s
Maximum load, kN	26	28	26	27.2
Maximum heating temperature, °C	157	195	208	231

**Table 4:** Chemical composition of points (in at.%).

	Cu	Zn	Ag
Point 1	2.55	96.30	1.15
Point 2	1.48	95.80	2.72
Point 3	0.73	92.03	7.24
Point 4	0.82	95.42	3.75

**Table 5:** Microstructural parameters from X-ray diffraction.

Processing	(AgCu)Zn_4_		AgZn_3_		Density of Dislocations, m^−2^
	Fraction, %	Size, nm	Fraction, %	Size, nm	
Rolling	2.22 ± 0.2	-	-	-	3.78 × 10^13^
ECAP 150 °C	15.34 ± 0.5	690	0.26 ± 0.06	90	11.74 × 10^13^
ECAP 200 °C	19.06 ± 0.7	720	0.78 ± 0.04	110	4.06 × 10^13^

**Table 6:** Thermal stability of microhardness of the ECAP samples, HV.

	20 °C	200 °C	250 °C	300 °C	350 °C
Initial	110 ± 20	99.7 ± 19.5	106 ± 12	106 ± 10	105 ± 21
ECAP Ø10 mm 150 °C	129 ± 9	116.8 ± 6	109 ± 10	124.8 ± 13	108 ± 15
ECAP Ø10 mm 200°C	111 ± 14	117 ± 7	115 ± 7	122 ± 11	116 ± 13

**Table 7:** Contributions from strengthening mechanisms.

Treatment	Calculated Data, MPa						Experimental Data, MPa	
	$\sigma_{{}_{o}}$	$\sigma_{{}_{\mathrm{gb}}}$	$\sigma_{{}_{d}}$	$\sigma_{{}_{\mathrm{ss}}}$	${\sigma_{\mathrm{pp}}}_{}$	${\Sigma \sigma }_{{}_{i}}$	UTS	YS
ECAP 150 °C	11	193	66	16	21	307	348	276
ECAP 200 °C	11	142	39	16	78	286	335	272

## Data Availability

Not applicable.
